# Thermal and Moisture Management Properties of Knitted Fabrics for Skin-Contact Workwear

**DOI:** 10.3390/ma18081859

**Published:** 2025-04-18

**Authors:** Simona Vasile, Jaime Paolo Vega Arellano, Cosmin Copot, Ahmad Osman, Alexandra De Raeve

**Affiliations:** 1 Fashion and Textiles Innovation Lab (FTILab+), HOGENT University of Applied Science and Arts, 9051 Ghent, Belgium; cosmin.copot@hogent.be (C.C.); alexandra.deraeve@hogent.be (A.D.R.); 2 School of Engineering, University of Applied Sciences in Saarbrucken, 66117 Saarbruecken, Germany; jaime.arellano@htwsaar.de (J.P.V.A.); or ahmad.osman@izfp.fraunhofer.de (A.O.); 3 Fraunhofer Institute for Nondestructive Testing IZFP, 66123 Saarbruecken, Germany

**Keywords:** personal protective clothing, skin-contact workwear, knitted fabrics, thermal and moisture management properties

## Abstract

Thermal and moisture properties of the textile materials worn in close contact with the skin greatly contribute to the comfort of the workwear and of the personal protective clothing (PPC) assemblies they are part of. This study examines in depth the thermoregulatory properties of eighteen knitted fabrics used in polo shirts and T-shirts, which function as thermal underwear, standard workwear compliant with various regulations, or as base layers in PPC systems. Most of the fabrics specifically engineered for heat protection demonstrated superior air permeability (ranging from 700 to 1200 mm/s) and efficient moisture management (OMMC 0.5–0.7). Their drying time varied between 12 and 18 min, comparable to most commodity fibre blend fabrics investigated. Generally, the heat-protective fabrics were heavier and exhibited greater thermal and vapour resistance. However, despite minor variations in predicted thermal comfort, seventeen of the fabrics were classified in the same cluster. These findings offer valuable insights into the thermal and moisture management properties of knitted fabrics with various levels of protection, and the correlations found between their thermoregulatory and physical properties, such as mass and thickness, provide guidance for the development of innovative knitted materials for workwear that enhance wearer comfort.

## 1. Introduction

There are multiple industrial and military situations in which workers are required to wear personal protective clothing (PPC) and equipment (PPE) [1]. The primary purpose of PPC is to protect workers from physical, mechanical, chemical, and other hazards. Workplace hazards range from low-risk exposures in industries like mining, oil and gas, building, and construction to high-risk scenarios such as firefighting, military operations, and electrical arc exposure [2].

By creating a barrier between the wearer and the environment, clothing interferes with the process of thermoregulation, particularly reducing dry heat loss and sweat evaporation. With increasing protection requirements, ergonomic problems also typically increase. These problems are often divided into thermal [3,4], metabolic [1,5,6], performance issues [7] and cognitive impairment or discomfort, fatigue, reduced manual performance, and injury [2,8].

A very important concern for workers wearing thermal protective clothing is its often detrimental effect on the wearer’s comfort. In addition to being potentially cumbersome, in order to protect sufficiently, the material of a garment must be substantial and is likely to add to the worker’s thermal load. The search for protective materials that also provide some level of comfort is long-standing and ongoing [9].

The importance of fibre composition and construction in heat and water vapour dissipation from the microclimate to the ambient environment was demonstrated earlier [10]. The selection of the fibres is dictated by workplace hazards, and the preference today is to use blends that take advantage of the beneficial properties of several fibre types [9,10,11].

Thermal and moisture management properties of textile fabrics for PPC have been investigated in many studies, with a focus on woven fabrics, with plain and twill weave constructions being the most common [11]. For example, ref. [12] reviewed design and materials for firefighters’ protective clothing, [13] reviewed both single-layer and multilayer fire protective clothing, and [14] proposed a categorization tool for fabric systems used in firefighters’ clothing based on their thermal protective and thermo-physiological comfort performances. Thermal and moisture management attributes of low-level risk woven fabrics were investigated by [11,15], who also compared them with civilian clothing materials and highlighted the potential to reduce fabric mass and thickness without compromising safety standards [2] and finally examined garment thermal attributes, construction, fit, and material characteristics [16]. An earlier study [17] also investigated the sensorial comfort of workwear woven fabrics and [18] addressed woven workwear fabrics for locomotor disabled employees. The study of [19] reviewed the protective and comfort performance of fire-protective textile materials used in workwear for firefighters and oilfield workers, identified knowledge gaps in the characterization and predictive modelling of fabric performance, criticized existing destructive, time-consuming, or expensive standardized testing methods, and stressed the need for models to predict performance based on physical properties of materials, aiming for improved safety and comfort. Similarly, ref. [20] provided a comprehensive review of several facets of clothing comfort, including assessment techniques and prediction models and proposed a conceptual comfort model that not only considers the thermal and evaporative resistance of the materials but, ideally, also additional fabric properties.

The thermal and moisture properties of the knitted textile materials worn in close contact with the skin also greatly contribute to the comfort of workwear and the PPC assemblies they are part of. Plenty of studies have addressed the comfort and quality of knitted fabrics in general [21,22] or next-to-the-skin (NTS) sportswear in commodity fibres [23,24,25]. A lot of research has been carried out to investigate the relationship between comfort and fabric structural parameters [26,27,28], fibre type [29,30,31,32,33,34,35], and finishing treatments of knitted fabric [36,37,38]. In addition to the assessment of the thermoregulation of fabrics, further studies considered wear trials to quantify the comfort of NTS garments and sportswear [39] or hybrid artificial intelligent approaches to predict global comfort of stretch garments [40] or thermal sensations, temperature, and skin wetness of sports underwear [41]. On the contrary, only a few studies were found that focus on knitted fabrics for skin-contact workwear in particular. Knitted fabrics can effectively transport moisture away from the skin, which is crucial for maintaining physiological comfort, especially in work environments where sweat accumulation can lead to discomfort and heat stress [42]. Comparative investigation [43] of thermal, mechanical, and comfort properties of heat-protective textiles recommended woven fabrics when strength is the primary concern and knitted fabrics when comfort is a priority. In high-risk occupations such as firefighting, poor moisture management can lead to severe discomfort and even injuries [19]. Large moisture quantities typically accumulate in the inner layer and underwear of firefighters’ protective clothing [44,45], and knitted underwear fabrics play an important role in transferring liquid sweat from the skin to the outer surface, keeping the skin dry and increasing the comfort level of the wearer [46]. The functionality and comfort of several knitted fabrics for firefighter underwear consisting of various FR and commodity fibre blends were investigated [47,48,49], and ref. [49] proposed knitted fabrics in blends of flame retardant (FR) viscose and merino wool as a viable economic alternative to pure aramid without compromising performance. Other studies [50,51] examined the influence of knitted structure and fibre composition on the thermo-physiological properties of heat-resistant protective workwear, and [52] investigated the influence of stitch types in single jerseys on the flammability and comfort of high-performance thermal clothing.

In short, the scientific literature is rich in studies investigating the comfort of woven fabrics for workwear and knitted fabrics in general. In contrast, the number of studies diving into the thermoregulatory properties of knitted fabrics for skin-contact workwear is rather scarce despite its paramount contribution to the thermal comfort of a PPC assembly. This study aims to create knowledge to fill this gap and examine in-depth thermoregulatory properties of a wide range of knitted fabrics designed for skin-contact workwear with various levels of protection. It investigates the relationship between the fabric structure and its thermoregulatory performance, applies established models from literature to predict their comfort performance, and uses statistical and cluster analysis for comparative evaluation and identification of groups with similar performance. The outcomes of this in-depth study should ultimately provide directions for designing knitted materials that enhance comfort in skin-contact workwear.

## 2. Materials and Methods

This study ultimately aims to develop an artificial neural networks (ANN) model to predict the comfort of single-layer, skin-contact workwear for predefined work intensity and environmental conditions. For this purpose, a large number of garments, differentiated by fibre composition and garment fit (loose and tight), are selected, and their comfort is being assessed through wear trials by healthy male volunteers executing an indoor treadmill running protocol, with monitoring of several subjects’ physiological body parameters.

### 2.1. Garment Selection

A total of thirteen T-shirts and five polos in various garment sizes and material composition were outsourced from leading international manufacturers and suppliers of workwear. All eighteen garments are worn by military, police, or workers in various industry sectors, in close contact with skin, as thermal underwear, workwear that complies with various standards or base layers in PPC assemblies. All the garments are listed in Table 1, which shows the garment description, garment purpose, and composition according to the manufacturer’s specifications. The fabrics are differentiated by fibre composition, ranging from natural and synthetic textile fibres (fabric ID 1, 3, 6, 7, 8, 11, 14, 15, 16, and 17), inherently or not-inherently flame retardant fibres (fabric ID 2, 9, 10, and 15), flame-resistant fibres (fabric ID 4, 5, and 18), or blends thereof [53]. Table 1 also displays images (magnification ×12) of the outer and inner side of the knitted structures, enabled by a ZEISS Stemi 508 stereo microscope (Carl Zeiss NV, Zaventem, Belgium) and a DeltaPix camera InVEnio 5SIII (DeltaPix, Smoerum, Denmark) with InSight 6.0 software for image processing.

### 2.2. Knitted Fabric Characterization

From each garment, samples were prepared, and physical and key thermo-physiological parameters of the knitted fabrics were assessed, following conditioning for at least 24 h in a controlled conditioning chamber at 65 ± 4% relative humidity and 21 ± 2 °C, according to EN-ISO 139:2005 [61].

#### 2.2.1. Physical Properties

Mass per unit area (g/m^2^) of the knitted fabrics was assessed according to ISO 3801:1977 [62], using a weight balance. Ten square specimens of 100 cm^2^ were weighted with an accuracy of 0.001 g, and the mean value and standard deviation (SD) were calculated.

The thickness of the fabrics (mm) was measured according to ISO 5084:1996 [63], under a pressure of 1 kPa, using thickness gauge Hess HDM-3 (Richard Hess MBV GmbH, Sonsbeck, Germany) with an accuracy of 0.01 mm. The mean value and SD of ten repeats were calculated to the nearest 0.1 mm.

The bulk density of the fabrics (kg·m^−3^) was calculated as the ratio of fabric mass per unit area (kg·m^−2^) and thickness (m). For the calculation of porosity, the following Equation (1) was used:

(1) $$P=\left(1-\frac{M}{\rho \times d}\right)\times 100,$$

where *M* is fabric mass per unit area (g·m^−2^), ρ is the fibre density (g·m^−3^), and *d* is fabric thickness (m). For instance, for a blend consisting of two components, the weighted average density of the fibre blend ρ was calculated depending on the fabric fibrous mixture using the equation below:

(2) $$\rho =a_{1}\times \rho_{1}+a_{2}\times \rho_{2},$$

where $a_{1},a_{2}$ and $\rho_{1},\rho_{2}$ are the fibre percentage in the blend and fibre density (g·cm^−3^), respectively [64].

The air permeability of the fabrics (mm/s) was assessed according to ISO 9237:1995 [65], using an air permeability instrument (EMI Development, Bréviandes, France) at a 100 Pa air pressure gradient and a 20 cm^2^ test area.

#### 2.2.2. Thermal and Moisture Management Properties

Key thermoregulation parameters of the knitted fabrics, including evaporative resistance, thermal resistance, moisture management, and drying time, were assessed.

Thermal resistance Rct (m^2^·°C/W) and water vapour resistance Ret (m^2^·Pa/W) were measured according to ISO 11092:2014 [66], using a sweating guarded hot plate, and the water vapour permeability index i*_mt_* (0–1) was calculated as ratio thermal and water vapour resistance: i*_mt_* = 60 × (Rct/Ret).

Moisture transport properties were assessed according to AATCC 195:2011 [67], using a Moisture Management Tester (MMT), SDL Atlas, Rock Hill, SC, USA. The MMT device provides a complete profile of next-to-the-skin worn fabrics by assessing their wetting time (WT), maximum wetted radius (MWR), spreading speed (SS), and absorption rate (AR), both on their top surface (T) and bottom surface (B). The accumulative one-way transport capability (R) and overall moisture management capacity (OMMC) were subsequently calculated, and the fabrics were categorized into seven categories. A grading of 1–5 (low–high) was applied to all indices. The mean value and SD were calculated for five specimens of 80 mm × 80 mm.

The drying time (min) was determined according to AATCC TM 201:2012 [68] using a DryRate^®^201 instrument from SDL Atlas, which combines a heating plate, a fan, and a high-precision temperature sensor. The instrument records the drying time of a sample horizontally placed on a metal plate preheated to 37 °C, wetted by 0.2 mL volume water droplet, and exposed to a horizontal airflow of 1.5 m/s. The water droplet is released from the bottom of the instrument on the inner side of the fabric in contact with the skin, and an infrared temperature sensor tracks fabric temperature changes, and determines when the fabric is fully dry. Three square samples (100 mm × 100 mm) were tested on each fabric, and the mean value and SD were calculated.

The thermo-physiological comfort performance (TCP) of the fabrics was determined using the model of [69], developed for fabrics used in workwear using the equation: TPC = 126.32 − 0.02 × W + 0.64 × SST − 0.05 × Ret, where Ret is evaporative resistance, W is fabric mass, and SST is water spreading speed on the top of the fabric, determined by MMT equipment.

Data analysis was performed using Python 3.10.2, calculating mean values and standard deviations (SD) to assess data distribution. Boxplots were used to visualise the distribution of properties like the fabric’s mass, thickness, bulk density, air permeability, drying time, drying rate, and OMMC, whereas bar plots represent the calculated variables (porosity, TCP, and i*_mt_*) or variables where only one specimen was tested (Rct and Ret). Since the data lacked normal distribution and potential nonlinear associations, a Spearman correlation was applied to assess the direction and strength of relationships among the tested properties. Correlations were classified as weak (r < 0.25), moderate (0.25 ≤ r < 0.75), and strong (r ≥ 0.75). All fabrics were analysed using ensemble clustering, combining K-means, hierarchical clustering, and DBSCAN methods to identify material similarities based on a co-association matrix, as suggested in [70]. Furthermore, the non-parametric Kruskal–Wallis test, ideal for cases where data are not normally distributed, used with post hoc comparisons to indicate significant differences among groups, allowed a systematic comparison of each fabric against all others to find similarities and differences in material properties, counting significant (*p* < 0.05) and non-significant results. A higher number of non-significant outcomes indicated greater similarity, while significant differences highlighted material variations, providing a comprehensive overview from a fabric properties perspective.

## 3. Results

The individual results of the measured physical and thermoregulation properties are displayed below as boxplots or bar charts. The fabrics are identified by the number of garments (as listed in Table 1), followed by fibre composition.

### 3.1. Physical Properties

**Figure 1:**
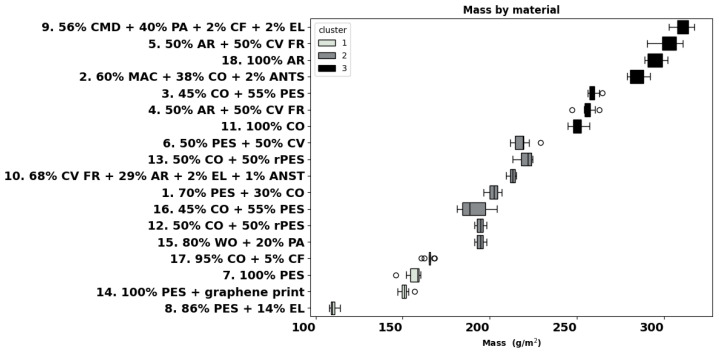
Mass per unit area of all materials (ISO 3801:1977).

Material mass influences garment weight and, therefore, is an important parameter for workwear, especially when it covers large body areas. The mass of the eighteen materials varied significantly, ranging from 109 g/m^2^ (fabric 8) to 310 g/m^2^ (fabric 9). Similarities among the materials were also observed with the three groups of the cluster analysis highlighted in different colours in Figure 1. Cluster 1 encompasses fabrics with a mass lower than 180 g/m^2^, including garments like 17, 7, 14, and 8. Cluster 2 includes fabrics 15, 12, 16, 1, 10, 13, and 6 with a mass ranging from 180 to 230 g/m^2^, and finally, cluster 3 encloses fabrics with a mass exceeding 230 g/m^2^ and consists of garments 11, 4, 3, 2, 18, 5, and 9.

Individual analyses of the fabrics showed that fabrics 6 and 10 differ from only one material (8 and 9, respectively), while fabrics 8 and 9 have the highest number of differences with the other garments. For instance, modal blend fabric 9 differs from fabrics 2, 3, 4, 5, 6, 9, 11, 13, and 18.

**Figure 2:**
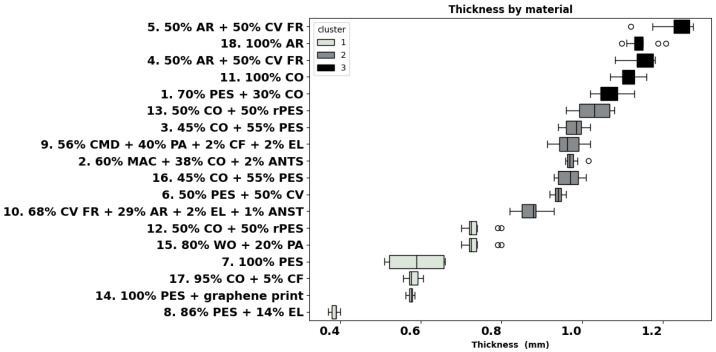
Thickness of all materials (ISO 5084:1996).

A material’s thickness is known to influence its thermal insulation and, therefore, heat transfer attributes. As can be seen in Figure 2, material 8 for sportswear is the thinnest of all (0.3 mm), and the aramid fabrics 4, 18, and 5 are thickest. Cluster analysis identified three groups, as illustrated in Figure 2. Cluster 1 includes fabrics with a thickness lower than 0.8 mm, such as fabrics 12, 15, 7, 17, 14, and 8. Cluster 2 ranges between 0.8 and 1.05 mm, with fabrics like 10, 6, 16, 2, 9, 3, and 13. Cluster 3 includes thickness greater than 1.05 mm, as it is for garments 1, 11, 4, 18, and 5. In individual analysis, materials 2, 3, 6, and 9 are similar to all other materials, except for material 8, and material 16 differs from material 5. Interestingly, fabric 8 for sportswear shows the highest number of differences (#10) with fabrics 1, 2, 3, 4, 5, 9, 11, 13, 16, and 18.

**Figure 3:**
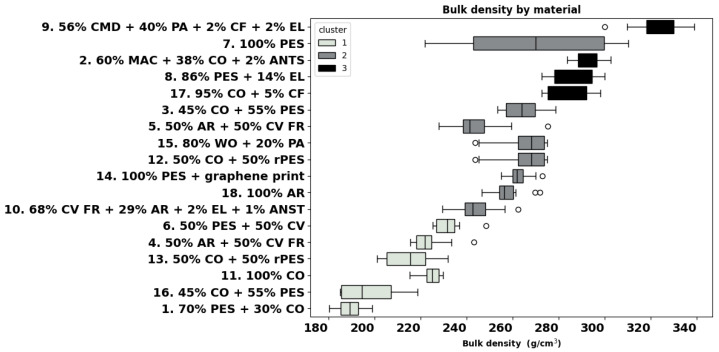
Calculated bulk density of all materials.

The bulk density of the knitted fabrics ranges from 189 g/cm^3^ (fabric 1) to 322 g/cm^3^ (fabric 9). Cluster analysis highlights three main groups with similar characteristics, as shown in Figure 3. Cluster 1 considers fabrics with a density lower than 233 g/cm^3^ with fabrics like 6, 4, 13, 11, 16, and 1. Cluster 2 ranges values from 234 to 270 g/cm^3^, including fabrics like 10, 18, 14, 12, 15, 5, and 3. In this cluster, a large data distribution can be noticed for polyester fabric 7, in line with its large thickness variation (Figure 2). Finally, cluster 3 includes garments with a density higher than 284 g/cm^3^, like fabrics 17, 8, 2, 7, and 9.

In the individual analysis, fabrics 5 and 10 show similarities with all materials, but both are different from garment 9. In contrast, the materials with the highest number of differences were fabrics 1 and 16, which differed from 2, 3, 7, 8, 9, 12, 14, 15, 17, and 18. Interestingly, these two fabrics share identical patterns of differences and similarities with other garments.

**Figure 4:**
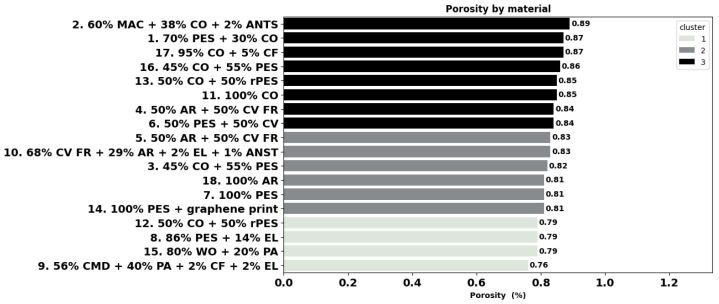
Calculated porosity of the materials.

Porosity refers to the proportion of open spaces (pores) within a fabric relative to its total volume. The calculated porosity of the fabrics is shown in Figure 4, indicating the modal fabric 9 and modacrylic blend fabric 2 as the least porous (76%) and most porous (89%), respectively. Cluster 1 consists of materials 12, 8, 15, and 9 with porosity lower than 80%. Cluster 2 consists of fabrics with a porosity between 81% and 83% and includes fabrics like 14, 7, 18, 3, 10, and 5. Finally, fabrics 6, 4, 11, 13, 16, 17, 1, and 2 were grouped in cluster 3 of fabrics with a porosity greater than 84%. Statistical tests for differences among materials were not executed due to insufficient data, as the porosity is a calculated parameter, as shown in Section 2.2.1.

**Figure 5:**
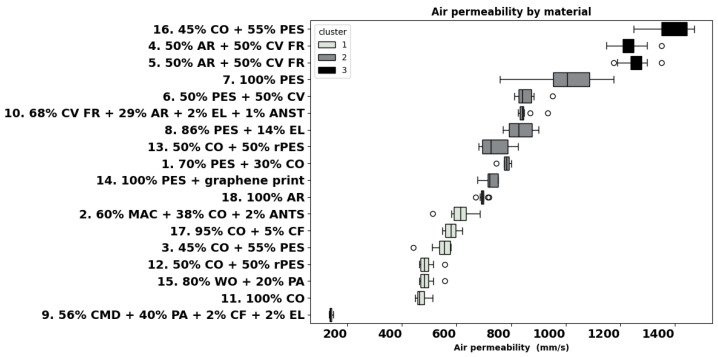
Air permeability of all materials (ISO 9237).

Greater air permeability of a material promotes convective cooling of the body, allowing wind and air movement to remove heat when a negative temperature gradient exists [2]. The air permeability measurements varied in a large range from 138 mm/s (fabric 9) to 1382 mm/s (fabric 16), as shown in Figure 5.

Cluster 1 consists of fabrics with air permeability lower than 613 mm/s, such as fabrics 2, 17, 3, 12, 15, 11, and 9. Fabrics 18, 14, 1, 13, 8, 10, 6, and 7, with an air permeability between 695 mm/s and 1011 mm/s, belong to cluster 2, and cluster 3 contains garments 5, 4, and 16 with air permeability superior to 1241 mm/s. The individual analysis indicated that garments 1 and 13 differ from material 9, while material 18 differs from 16. Notably, fabrics 9 and 16 have the highest number of differences (#9) across all materials.

### 3.2. Thermoregulation Properties

The dry heat, water vapour transfer and liquid moisture transfer attributes of materials are important factors in thermal management and, therefore, wearer comfort. The lower the resistance to dry heat and water vapour transfer, the more easily heat and vapour can pass from the inside of the workwear to the ambient environment. When hot conditions combine with high metabolic work activities, sweating creates a need for efficient transfer of liquid moisture [2].

**Figure 6:**
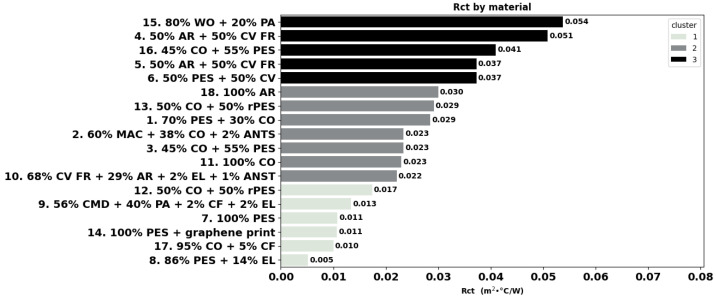
Thermal resistance Rct of the fabrics (ISO 11092).

The thermal resistance of all the fabrics is displayed in Figure 6, which shows that among all fabrics, the polyester blend fabric 8 has the lowest thermal resistance Rct value of 0.0052 m^2^·°C/W. In contrast, aramid blend fabric 4 has a ten times higher thermal resistance (0.051 m^2^·°C/W), and the wool blend 15 slightly exceeded it, which makes it suitable as thermal underwear for cold protection. The polyester blend fabrics 7, 8, and 14 exhibit the lowest Rct values, recommending them for sport or intense activity work. Three clusters were identified, where cluster 1 consists of fabrics 12, 9, 7, 14, 17, and 8 with a thermal resistance lower than 0.018 m^2^·°C/W. Cluster 2 covers moderate thermal resistance ( 0.022 and 0.030 m^2^·°C/W) fabrics 10, 11, 3, 2, 1, 13m and 18 and cluster 3 contains fabrics 6, 5, 16, 4, and 15 with a thermal resistance higher than 0.037 m^2^·°C/W.

In Table 2, a significant moderate positive correlation of Rct with fabric’ thickness (r = 0.63, *p*-value < 0.05) can be seen, as well as a significant negative correlation with bulk density (r = −0.56, *p*-value < 0.05), suggesting that thicker materials tend to have higher thermal resistance, whereas denser materials demonstrate lower resistance.

In line with the correlations above, thicker polo fabric 13 exhibits a higher Rct value than T-shirt fabric 12 with the same composition (50% CO + 50% rPES). However, this is not the case for the undershirt for cold protection fabric 3, which has a lower Rct and performs better than fabric 16 (both 45% CO + 55% PES) despite its higher thickness and mass. Similarly, slightly thicker and heavier aramid fabric 5 has a lower Rct and thus performs better than fabric 4 with the same aramid blend. With a similar thickness, higher porosity, and air permeability, polo fabric 16 shows a higher Rct value than fabric 3 in the same blend (45% CO + 55% PES blend). Polyester fabrics 7 and 14 with similar thicknesses also exhibit comparable Rct. Our findings largely align with those of [2], particularly for correlations involving thickness and bulk density. However, some discrepancies appear in regards to the significance of relationships for Rct versus mass and air permeability. The direct comparison with [71] results is ambiguous due to limited comparison in their publication.

**Figure 7:**
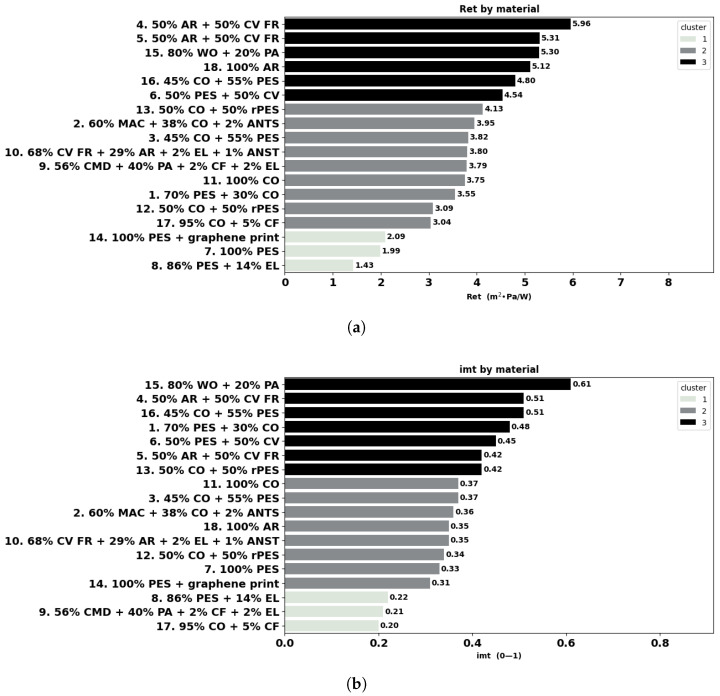
Vapour resistance Ret (**a**) and permeability index i*_mt_* (**b**) of the fabrics (ISO 11092:2014).

The water vapour resistance largely varied between 1.43 m^2^·Pa/W for polyester fabric 7 and 5.96 m^2^·Pa/W in the case of aramid-viscose FR fabric 4, as can be seen in Figure 7a. Cluster analysis highlights three main groups. Cluster 1 includes garments with a resistance lower than 2.1 m^2^·Pa/W with polyester fabrics for sportswear garments (14, 7, and 8), suitable for high-intensity sports activities. Cluster 2 includes garments 17, 12, 1, 11, 9, 10, 3, 2, and 13 with a thermal resistance between 3.04 m^2^·Pa/W and 4.13 m^2^·Pa/W. Finally, cluster 3 includes fabrics 6, 16, 18, and 15 and aramid blends 5 and 4 with values higher than 4.5 m^2^·Pa/W.

As shown in Table 2, Ret displays a significant positive correlation with thickness (r = 0.68) and mass (r = 0.61), indicating that thicker and heavier materials have higher water vapour resistance, which is in agreement with the study of [41]. Our findings are also in line with those of [2], who found the same correlations between Ret and fabric thickness, mass, and bulk density. In agreement with the correlations found, thicker and heavier polo fabric 13 has a higher Ret-value than T-shirt fabric 12, which has the same composition (50% CO + 50% rPES). Similar thickness, higher porosity and air permeability polo fabric 16 show a higher Ret-value than the undershirt for cold protection fabric 3, which are both a blend 45% CO + 55% PES. Slightly heavier and thicker aramid fabric 5 has a comparable Ret-value with fabric 4 in the same composition, and polyester fabrics 7 and 14 with similar physical parameters also exhibit comparable Ret.

The water-vapour permeability index i*_mt_* is dimensionless and has values between 0 and 1, where a value of 0 implies that the material is water-vapour impermeable, while a material with a value of 1 has both the thermal resistance and water-vapour resistance of an air layer of the same thickness. The results in Figure 7b indicate the highest i*_mt_* value (0.61) for fabric 15 (80% WO + 20% PA). Cluster 1 includes fabrics 8, 9, and 17 with an i*_mt_* lower than 0.22. Cluster 2 includes fabrics 14, 7, 12, 10, 18, 2, 3, and 11 with values between 0.31 and 0.37 and finally, fabrics 13, 5, 6, 1, 16, 4, and 15 with i*_mt_* greater than 0.42 are grouped in cluster 3. As shown in Table 2, the water vapour permeability index is significantly positive and negatively correlated with fabric thickness (0.54) and bulk density (−0.69), respectively. The relationship between i*_mt_* and fabric mass in our study is weak positive but not statistically significant (r = 0.21), which contrasts with the strong negative correlation observed by [71]. Our results indicate a positive but non-significant correlation (r = 0.34) between i*_mt_* and porosity which is consistent with their findings but differs in the strength and significance of the association. On contrary, [41] found a negative non-significant correlation between i*_mt_* and air permeability and a significant strong negative correlation with fabric thickness.

Moisture management properties were assessed by the Moisture Management Tester (MMT). The mean values (SD) of the eight moisture management attributes of eighteen materials are summarized in Table 3.

In general, despite some large standard deviations observed in the case of fabrics 12, 7, 9, and 2, the majority of the fabrics in conventional fibre blends (11, 12, 13, 3, 16, 6, 1, 8, 7, 14, and 15) have lower moisture spreading speed SS*_T,B_* (below 1 s) than the high thermal and heat protection fabrics (17, 9, 2, 10, 4, 5, and 18). Among all fabrics, moisture management fabric 8 (86% PES + 14% EL) has the shortest wetting time WT*_T,B_*, the fastest moisture spreading speed SS*_T,B_*, and the largest wetted radius WR*_T,B_*. Within the group of fabrics for thermal and heat protection, aramid fabric 18 has the fastest SS*_T,B_*, followed by fabrics 5 and 10 (both AR and CV FR blends), and all three fabrics were characterized as moisture management fabrics. These three fabrics also showed the shortest wetting time WT*_T,B_*. On the contrary, water penetration fabrics 4 (50% AR + 50% CV FR) and 17 (95% CO + 5% CF) exhibited a zero SS*_T_*, as the moisture was instantly transported at the bottom surface. For that reason, these two fabrics also did not get wet on the top (skin side), as shown by the high values of the WT*_T_* and zero value wetted radius MWR*_T_*. A similar situation was observed for the water penetration fabrics 13 and 16, but, in a lesser manner, also for the moisture management fabric 1, all three blends of polyester and cotton.

As shown in Table 2, moisture management parameters are correlated with several physical parameters of the fabrics. For instance, fabric bulk density is significantly negatively correlated with wetting time WT*_T_* (r = −0.38), in agreement with [2] and positively with MWR*_T_* (r = 0.42) and MWR*_B_* (r = 0.29), differing from their findings. In addition, this fabric property was positively moderate (r = 0.45) and weakly positive (r = 0.2) correlated with the spreading speed on the top of the fabric (SS*_T_*) and on the bottom of the fabric (SS*_B_*), respectively. Similarly, fabric mass demonstrated significant positive and negative correlations with SS*_T_* (r = 0.44) and SS*_B_* (r = −0.22), respectively. It also correlated negatively with WT*_T_* (r = −0.30) and positively with WT*_B_* and MWR*_T_* (both r = 0.36). Air permeability showed significant moderate negative correlations with SS*_T_* (r = −0.32, *p* < 0.05), WT*_B_* (r = −0.23, *p* < 0.05), and MWR*_T_* (r = −0.25, *p* < 0.05) and moderate positive correlations with WT*_T_* (r = 0.25, *p* < 0.05). Fabric thickness was significantly negatively correlated with SS*_B_* (r = −0.21, *p* < 0.05) and MWR*_B_* (r = −0.24, *p* < 0.05) and showed no correlations with SS*_T_*, WT*_T_*, WT*_B_*, and MWR*_T_*. Finally, porosity had a significant moderate negative correlation with SS*_T_* and MWR*_T_* (both r = −0.55) while showing a positive moderate correlation with WT*_T_* (r = 0.47).

In line with these correlations, fabric 12, with a higher bulk density, has a lower wetting time WT*_T_* (64 s) than fabric 13 (120 s) in the same composition (50% CO + 50% rPES), and also its wetted radius MWR*_T,B_* is higher. The same was observed for denser fabric 3 (45% CO + 55% PES), which displayed lower WT*_T_* and higher wetted radius MWR*_T,B_* (20/15 mm) than fabric 16 (3/6 mm) and also for aramid fabrics 5 and 4. An exception was the bulkier fabric 7, which has both a higher WT*_T,B_* and MWR*_T,B_* than fabric 14 in the same composition, but this may be explained by the large SD of the bulk density of fabric 7.

**Figure 8:**
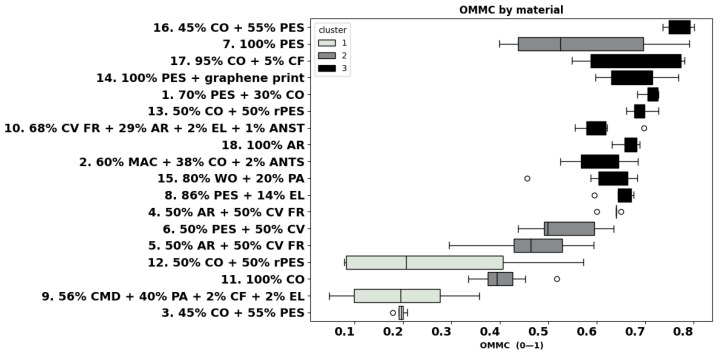
OMMC values of all the fabrics.

As shown in Figure 8, most of the fabrics exhibited a high OMMC in the range of 0.5–0.7, with fabric 16 (45% CO + 55% PES) even reaching an OMMC value of 0.8. Cluster analysis highlights three groups, where cluster 1 reflects the poorest OMMC grade with a maximum value of 0.19 and includes fast-absorbing, quick-drying fabric 3 and slow-absorbing, slow-drying fabrics 9 and 12. Cluster 2 contains fabrics 11, 5, 6, and 7 with OMMC values between 0.46 and 0.53; cluster 3 shows higher values than 0.61, including water penetration fabrics 2, 13, 16, and 17 and water management fabrics 16, 17, 14, 1, 13, 10, 18, 2, 15, and 8. Water penetration fabric 4 also belongs to this cluster, probably due to its large variation and many outliers.

Our results show partial agreement with the findings of [71]. As shown in Table 2, we observed a negative but not statistically significant correlation between OMMC and thickness (r = −0.07, *p*-value > 0.05). However, our results indicate a significant negative correlation between OMMC, bulk density (r = −0.33), and mass (r = −0.4), and also a moderate positive correlation with air permeability (r = 0.5). While this supports their claim that density affects moisture transfer, the correlation strength in our study is rather moderate. In line with the correlation found, for the same composition, heavier fabrics 12, 3, and 7 have lower OMMC than the fabrics 13, 16, and 14, respectively.

**Figure 9:**
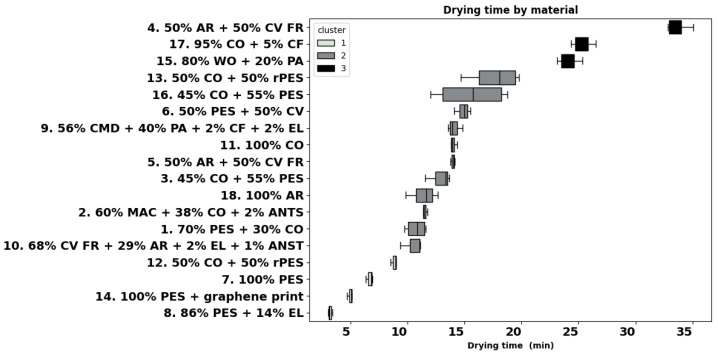
Drying time of the fabrics (AATCC TM 201:2012).

The drying time of the eighteen fabrics, laid on a metal plate at 37 °C, exposed to an airflow of 1.5 m/s applied parallel with the fabric surface, was measured after the surface was wetted with 0.2 mL of water. Most of the fabrics dried within 5–15 min; thin fabric 8 (86% PES + 14% EL) dried quickest in about 3 min, and underwear fabrics 15 (80% WO + 20% PA) and 17 (95% CO + 5% CF) dried in roughly 25 min. Fabric 4 (50% AR + 50% CV FR) dried slowest (33 min). Cluster analysis highlighted three groups, as shown in Figure 9. Cluster 1 includes fabrics 12, 7, 14, and 8, which dried in less than 9 min, and cluster 2 includes fabrics with a drying time between 10 and 18 min, namely fabrics 10, 1, 2, 18, 3, 5, 11, 9, 6, 16, and 13. Finally, aramid fabric 4, cotton blend fabric 17, and wool fabric 15, which had the longest drying times (more than 24 min), were grouped in cluster 3.

As shown in Table 2, drying time shows a positive and statistically significant relationship with fabric mass (r = 0.35) and thickness (r = 0.41), suggesting that heavy and thick fabrics tend to dry slowly. In line with these correlations, heavier and thicker polo fabric 13 (50% CO + 50% rPES) dried slower than T-shirt fabric 12 in the same blend. The same trend was observed for the polyester fabrics 7 and 14. Surprisingly, lighter polo fabric 16 dried slower than the undershirt for cold protection fabric 3 in the same blend (45% CO + 55% PES blend), but this can be attributed to the large SD observed for fabric 16. Similarly, the lighter aramid fabric 4 dried much slower (33 min) than fabric 5 (both a blend of 50% AR + 50% CV), and they also belong to different clusters.

In addition, Table 4 highlights significantly positive correlations between the drying time, Ret (r = 0.63), and Rct (r = 0.58), suggesting that fabrics with greater thermal and moisture resistance take longer to dry. Our findings are partially aligned with the study of [41] that reported even stronger significant positive and negative correlations of dying time with Ret and imt, respectively. Drying time negatively correlated with MWR*_B_* (r = −0.65, *p*-value < 0.05) and SS*_B_* (r = −0.59, *p*-value < 0.05), showing that faster moisture spreading and higher moisture radius on the external face contribute to faster drying of the fabric, and it was positively correlated with WT*_T_* (r = 0.47, *p*-value < 0.05) and with SS*_B_* negative (r = −0.59, *p*-value < 0.05). These results reinforce the importance of thermal and structural fabric properties in determining drying behaviour, as also shown in [72].

**Figure 10:**
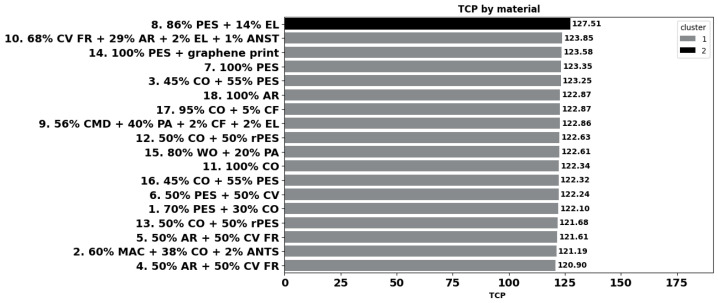
Thermo-physiological comfort performance TCP of the fabrics.

The thermo-physiological comfort performance (TCP) of the fabrics was assessed using the model of [69], developed for single-layered and multi-layer woven fabrics used for workwear, and includes key fabric parameters such as mass, moisture spreading speed, and evaporative resistance. As illustrated in Figure 10, the TCP of the eighteen fabrics varied between 120 and 127, and the cluster analysis highlighted two groups where the main difference was with fabric 8, showing the highest TCP score among all fabrics.

The results indicate fabric 8 (86% PES + 14% EL) as the most comfortable, which was shown by its good thermoregulatory properties, and this was also confirmed by the results of our first wear trials, with five male test subjects with a mean age of 24 ± 2 years. They executed a running treadmill protocol at a constant speed of 8 km/h for a total duration of 30 min in a predefined indoor environment of temperature 21 ± 1 °C and humidity RH 46 ± 8 %. On a five-point comfort scale (−4 to 0), more than 80% of participants indicated a T-shirt in fabric 8 as the most comfortable. On the contrary, fabric 4 (50% AR + 50% CV FR) seems to be the least comfortable. The results of the wear trials are in progress, and a manuscript with details about the protocol and the results is being prepared.

## 4. Discussions

### 4.1. Fabric Mass

Direct comparison of the eighteen knitted fabrics shows, with small exceptions, a higher mass for the high thermal and heat protection fabrics, with fabric 9 (56% CMD + 40% PA + 2%CF + 2% EL) being the heaviest, followed by aramid fabrics 5 and 18 and modacrylic-cotton blend fabric 2. With the exception of fabric 17 (90% CO + 5% CF), the lightest fabrics in the cluster of 100–180 g/m^2^ were fabrics 8, 7, and 14 for sportswear. Most of the fabrics were in the range of 180–230 g/m^2^, and the majority of fabrics for thermal and fire protection (4, 2, 18, 5, and 9) exceeded 230 g/m^2^ and were grouped in the same cluster, together with polo fabric 11 of 100% cotton. This increase in mass reflects the higher mandatory protection requirements but also a difference in the knitted structure, with the fabrics for polos being, in general, heavier than those for T-shirts.

### 4.2. Fabric Thickness

Similarly, the group of fabrics for thermal and heat protection were significantly thicker than the rest of the fabrics, the majority being classified in cluster 2 and fabrics 4, 18, and 5 in cluster 3. The correlations found indicate that a reduction in mass and thickness provides lower thermal resistance, which is important for hot conditions. A similar trend was observed for vapour resistance.

### 4.3. Air Permeability

Air permeability refers to the ability of a fabric to allow air to pass through it. High air permeability enhances breathability, making the fabric more comfortable in hot and humid conditions [21]. Among polyester fabrics 7, 8, and 14 for sportswear, fabric 7 seems to have the highest mean value for air permeability but also the largest variability SD. These three fabrics are lightweight, have low thermal resistance, and dry quickest, which shows their suitability for sports activities. Among the fabrics used for underwear and base layers, the aramid-CV FR blends 4 and 5 exhibited a high air permeability of around 1200 mm/s, and undershirt fabric 16 (45% CO + 55% PES) was the most permeable of all fabrics. The values found are in agreement with [48], where air permeability values between 1600–1800 mm/s were reported for two-layer knitted structures for underwear in aramid and aramid-CV FR blends. Within the group of fabrics for thermal and heat protection, tight-knitted modal blend fabric 9 had the poorest air permeability. Positively, aramid blend fabrics 4 and 5 belong to the cluster with the highest permeability (>900 mm/s), and aramid blend fabrics 18 and 10 belong to the second cluster (700 and 900 mm/s).

### 4.4. Thermal Properties

Thermal resistance measures how well a fabric can insulate against heat. Fabrics with high thermal resistance are ideal for cold environments, while low-resistance fabrics are suitable for warm conditions [43]. The fabrics for thermal protection underwear in this study exhibited the highest Rct value. Within this group, wool blend fabric 15 has the highest Rct value and potentially isolates best against cold, followed by aramid-CV FR fabrics 4 and 5, PES-CV blend fabric 6, and PES-CO blend fabric 12. Positively, polyester fabrics 7, 8, and 14 for sportswear exhibit the lowest Rct value among all fabrics. Comparable polyester fabrics 14 and 7 have a similar Rct, which indicates that the graphene print applied on fabric 14 does not influence its thermal resistance. This is in disagreement with [73], who showed the benefits of blade-coated graphene on the thermal conductivity of cotton fabrics for welding protective clothing, and [74] claimed graphene and workwear as a winning combination for cooling and comfort. Manufacturers of graphene-printed fabrics comparable with fabric 14 also claim body cooling due to the distribution of heat along the graphene circuit in contact with the skin. The study of [75] also reports some differences in the thermal and moisture properties of the fabrics depending on the design of the graphene pattern. They comparatively investigated the thermal properties of polyester fabrics with two distinctive graphene patterns using a Fabric Touch Tester (FTT) device and by thermal imaging. Similarly to the hot plate method used in this study, the FTT records the maximum thermal flux Qmax that transversally passes through fabrics during a test period of 120 s. Nevertheless, the results of thermal imagining clearly demonstrated an almost instant dissipation of heat flux along the graphene circuit and not transversally through the fabric thickness. In line with their findings, the instrumental method used in this study seems not suitable to assess the thermal properties of this fabric. Therefore, the potential body cooling sensation of this T-shirt will be further investigated via wear trials with test subjects.

### 4.5. Water Vapour Permeability

Water vapour permeability measures how well a fabric can transmit moisture vapour. High water vapour permeability helps in maintaining a comfortable microclimate between the skin and the fabric [21]. The ability of the body to transfer heat by evaporation of vapour and liquid moisture from the skin provides an opportunity for large amounts of heat to dissipate [76]. The primary way to increase heat loss in hot environments is to reduce the thermal and evaporative resistance of the materials worn against the skin, which creates a barrier between the skin and the environment. Unfortunately, frequent consequences of the external protection requirements are that workwear fabrics resist heat loss due to low permeability to water vapour, reducing heat transfer and cooling and, therefore, increasing the risk of heat stress [49]. Concerning the materials that belong to the group of heat and thermal protection, aramid blends 4 and 5 in particular also had the highest resistance to water vapours, as opposed to the polyester fabrics for sportswear, which performed best, with fabric 8 (86% PES + 14% EL) in particular.

Thickness appears to play an important role in both the thermal (Rct) and water vapour (Ret) resistance of the fabrics; bulk density has relevance only for Rct. These correlations provide directions in fabric development, indicating that a reduction in fabric mass and thickness provides lower vapour resistance, which is important for hot conditions.

### 4.6. Liquid Moisture Properties

The liquid moisture transfer properties of materials become critical in hot conditions where sweat is formed quickly, and high sweating rates result from work activities. In these circumstances, moisture transfer becomes more important than Ret [2]. Wet knitwear results in a significant loss of comfort [77]; therefore, fabrics which can quickly absorb and transport moisture away from the skin keep the wearer dry and comfortable [21]. Comfort is positively related to perceived warmth and negatively to perceived dampness; an increase in the moisture in the skin–clothing microclimate decreases the wearer’s perception of comfort [78]. There is a strong relationship between increased wetness perception and reduced thermal comfort [79]. When humans are in warm ambient conditions, skin wetness is more strongly related to perceived thermal comfort than to the skin’s surface temperature [80]. Previous studies [45,46] highlighted the important contribution of inner layers and underwear in overall comfort perception, showing that at least 75% of moisture remains in the clothing’s layers, especially in the underwear and inner layer [45] of firefighters’ protective clothing. The study of [44] detected between 50% and 80% of the sweat accumulated in the inner two layers, depending on the fibre composition investigated, among which were wool, cotton, and aramid. In our study, within the group of garments with fabrics 4, 5, 6, 12, 15, and 16 worn as underwear or a baselayer in a PPC assembly, the OMMC varied largely between 0.2 (fabric 12, 50% CO + 50% rPES) and 0.7 for fabric 16 (45% CO + 55% PES). Fabric 12 was classified as a slow-absorbing, slow-drying fabric and fabric 16 showing water penetration, along with aramid fabric 4 (OMMC 0.63), meaning that they both quickly transport sweat from the skin side keeping skin dry. Wool fabric 15 exhibits a high OMMC above 0.6, and it is classified as a moisture management fabric. Our results are in agreement with [48], which reported high OMMC values in the range of 0.6–0.8, depending on the knitted structure of aramids and aramid-CV FR underwear. The study of [49] concluded that blends of CV FR and merino wool are viable alternatives to pure aramid underwear in terms of performance, cost, and comfort. Polyester fabrics for sportswear 7, 8, and 14 have OMMC values exceeding 0.6 and are all classified as moisture management fabrics.

### 4.7. Drying Time

Fabrics that quickly dry are preferred, as moisture accumulated in the inner layer leads to wearer discomfort, as was established. Most of the fabrics in this study dried in intervals of 10–15 min, with sport polyester fabrics 7, 8, and 14 drying the quickest, which is a very positive factor during intense sports activities. Among the group of base layer garments 4, 5, 6, 12, 15 and 16, the drying time varied largely between 8 min (fabric 12, 50% CO + 50% rPES) and 33 min for aramid fabric 4. Surprisingly, fabric 12 exhibits a low OMMC (0.2), and it was classified as a slow-absorbing, slow-drying fabric. Despite its long drying time, aramid fabric 4 is a water penetration fabric, which quickly transports sweat from the skin to the next layer of clothing and keeps the skin dry, which makes this fabric suitable as an undergarment. The same aramid blend, heavier fabric 5, dried about 60% faster and was classified as a moisture management fabric. Within the group of fabrics for heat and thermal protection, with a low mean value of OMMC and large SD, modal fabric 9 scored the worst, and it was classified as a slow-absorbing, slow-drying fabric. All the other fabrics within this group showed a high OMMC with values between 0.5 and 0.7.

Fibres that absorb more moisture, i.e., natural fibres like cotton, dry slower than synthetics [10,11]. In our case, underwear fabrics 6 (50% PES + 50% CV) and 16 (45% CO + 55% PES) had a comparable drying time of around 15 min, much lower than the wool fabric 15 (24 min). Apart from fabric 12 (50% CO + 50% rPES), none of the blends of cotton, viscose, or viscose FR dried faster than 10 min, which is in agreement with the study of [10], where among all synthetic and natural fibre fabrics investigated, the longest drying time was found for the 100% cotton fabric. In the case of similar structures, these results can be linked to higher moisture regain of CO (8.5%), CV (11%), and WO fibres (16%) as opposed to synthetic fibres such as PES (0.4%) or MAC (1.5%) (ASTM D1909) [72,81].

### 4.8. Thermal Comfort Performance

The calculated thermal comfort performance following the model of [69] clearly indicated fabric 8 (86% PES + 14% EL) for sportswear as the most comfortable. This fabric also displays excellent thermoregulatory properties, which was also confirmed by the results of our first wear trials. Within the group of fabrics for thermal and heat protection, fabric 4 (50% AR + 50% CV FR) seems to be the least comfortable of all eighteen fabrics. The best fabric in this group appears to be fabric 10 (68% CV FR + 29% AR + 2% EL + 1% ANST), followed by fabric 17 (95% CO + 5% CF), fabric 9 (56% CMD + 40% PA + 2% CF + 2% EL), and fabric 18 (100% AR), which all overperformed most of the fabrics in commodity fibre blends, except sport polyester fabrics 7, 8, and 14 and fabric 3 (45% CO + 55% PES). Though developed for woven fabrics, the prediction of this model also seems to be suitable for knitted fabrics, and it was partially validated by its own wear trials. Similar models are valuable tools for designers and manufacturers, supporting them in the development of workwear with elevated thermoregulatory properties, with minimum testing.

**Figure 11:**
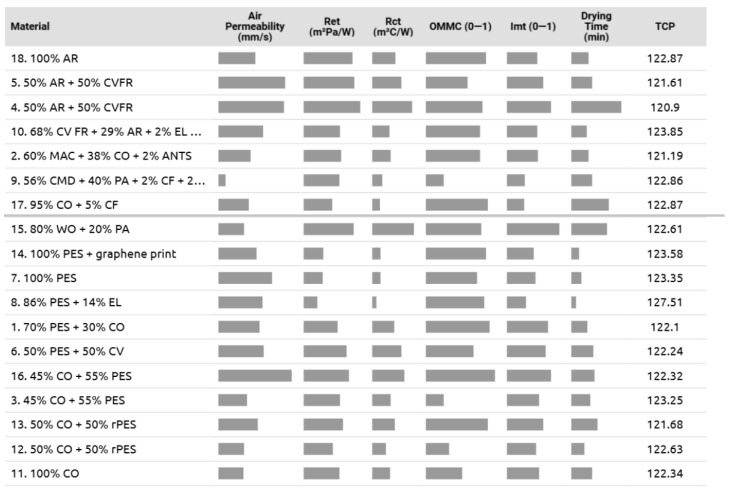
Summary of the thermal and moisture properties of the knitted fabrics: longer bars for air permeability, OMMC, and i*_mt_* and shorter bars for Ret, Rct, and drying time, respectively, perform best. High scores for the TCP indicate high predicted thermo-physiological comfort performance.

Finally, all the fabrics are ranked, starting with 100 % cotton fabric 11 and ending with 100 % aramid fabric 18, and all thermoregulation properties investigated are displayed in Figure 11 to clearly show the combination of parameters that lead to their thermal comfort performance. For instance, the most comfortable polyester fabric 8 for sportswear has good moisture properties, dries fast, and has low thermal and evaporative resistance. In contrast, fabric 4 (50% AR + 50% CV FR), which has high thermal and vapour resistance and the highest drying time, was predicted as the least comfortable.

Within the group of thermal and heat protection, fabric 10 (68% CV FR + 29% AR + 2% EL + 1% ANST) was indicated as the most comfortable fabric, and it has good moisture management properties, low thermal and vapour resistance, and a short drying time. A good choice is also aramid fabric 18, but its air permeability can be improved, similar to fabric 9 (56% CMD + 40% PA + 2% CF + 2% EL), which also has poor moisture management properties.

Despite similar air permeability and moisture management properties, the group of thermal and heat protection fabrics 18, 5, 4, 10, 2, 9, and 17 (situated above grey horizontal line in Figure 11) exhibits average higher thermal and vapour resistance, lower permeability to vapours, and a longer drying time, and it can be therefore assumed that the wearers would perceive them as less comfortable, in comparison with the rest of the fabrics.

## 5. Conclusions

The thermal and moisture properties of materials worn in close contact with the skin greatly contribute to the comfort of single-layer workwear and of the PPC assemblies they are part of. This study conducted an in-depth analysis of the thermoregulatory properties of knitted fabrics in various structures and fibre blends that comply with various levels of protection. The findings presented in this paper provide insights and quantification of the thermoregulation properties of knitted fabrics used in workwear in particular. The results, based on a significant number of fabrics investigated, clearly demonstrate the complexity of developing comfortable materials for workwear due to a multitude of relevant influencing factors, among which were fibre blend and several structural parameters, which all affect the thermoregulatory properties of the fabrics in different manners, though not always statistically significant. The cluster analysis and correlations found provide valuable insights into groups of fabrics with comparable properties and should offer directions for designing knitted materials that enhance comfort in skin-contact workwear. A prediction model, as used in this paper, can be implemented in the textile industry and academia to conveniently predict thermo-physiological comfort performance by utilizing key fabric parameters. It is expected to simplify and reduce the cost of the fabric development process. Although this model was not specifically developed for knitted fabrics, the results are in agreement with the findings of our preliminary wear trials. However, it can be further refined by including some additional key fabric parameters and the dataset of knitted fabrics should be extended further. Further work will validate the comfort performance of the fabrics using the established running protocol. The study’s ultimate goal is to develop an ANN model to predict the thermal, moisture, and overall comfort sensations of knitted fabrics for single-layer garments in specific use scenarios. Model validation will incorporate data from participants and multiple sensors and combine subjective and objective measurements. This validation will support clothing manufacturers, particularly in the workwear and sportswear sectors, in designing garments with enhanced thermal comfort.

## Figures and Tables

**Table 1:** Description of the selected garments, fabric blend composition, and images of the outer and inner fabric structure (magnification ×12); PES-polyester; CO-cotton; CV FR- fire-retardant viscose; AR-aramid; MAC-modacrylic; CMD-modal; WO-wool, PA-polyamide; rPES-recycled polyester; ANST- Acrylic Nitrile Staple; EL-elastane; CF-carbon (ISO 2076 [54]; ISO 6938:2012 [55]).

	Garment Description and Destination	Fabric Composition	Fabric Structure	
			Outer	Inner
1	Short-sleeved polo; police shooting monitors	70% PES + 30% CO	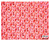	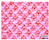
2	Short-sleeved polo; maritime police; compliant with ISO 11612 [56] (A1B1C1) and EN 1149-5 [57]	60% MAC + 38% CO + 2% ANST	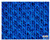	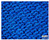
3	Short-sleeved polo; basic clothing, police	45% CO + 55% PES	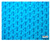	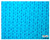
4	Long-sleeved T-shirt; winter undergarment compliant with ISO 11612, police	50% AR + 50% CV FR	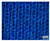	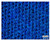
5	Long-sleeved T-shirt; summer undergarment compliant with ISO 11612, police	50% AR + 50% CV FR	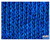	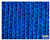
6	Long-sleeved T-shirt; Thermal underwear, police	50% PES + 50% CV	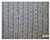	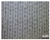
7	T-shirt; standard sportswear, police	100% PES	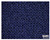	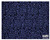
8	T-shirt; standard sportswear	86% PES + 14% EL	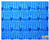	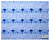
9	Long-sleeved seamless T-shirt compliant with ASTM D6413/D6413M-15 [58]; military	56% CMD + 40% PA + 2% CF+ 2% EL	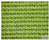	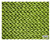
10	Long-sleeved seamless T-shirt compliant with ISO 14116 [59] (FR index 3); military, firefighters	68% CV FR + 29% AR + 2% EL + 1% ANST	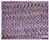	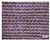
11	Short-sleeved polo, compliant with the ASTM D6413/D6413M-15; military	100% CO	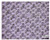	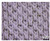
12	Short-sleeved T-shirt; base layer in industrial and healthcare sector	50% CO + 50% rPES	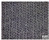	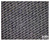
13	Short-sleeved polo; industrial and healthcare sector	50% CO + 50% rPES	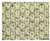	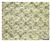
14	Long-sleeved T-shirt with thermoregulation properties; industrial and healthcare sector	100% PES graphene print inside	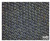	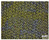
15	Long-sleeved T-shirt; winter thermal underwear worn under the combat uniform, military	80% WO + 20% PA	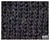	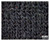
16	Long-sleeved T-shirt; undershirt, cold protection, and industrial applications, i.e., cool chain logistics, food industry, etc.	55% PES + 45% CO	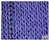	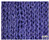
17	Long-sleeved polo; compliant with EN 61340-5-1-2016EDS [60] protection, automotive, electronics, etc.	95% CO + 5% CF	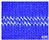	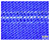
18	Long-sleeved turtleneck; military	100% AR	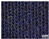	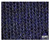

**Table 2:** Spearman’s correlation coefficients between some thermal and textile properties: thermal resistance Ret, water vapour resistance Ret, water vapour permeability index i*_mt_*, overall moisture management coefficient OMMC, water spreading speed SS, wetting time WT, and MWR maximum wetted radius on the top (T) and bottom (B) of the fabric. Values in bold with * indicate significant correlations (*p*-value < 0.05).

	Rct	Ret	i*_mt_*	OMMC	SS*_T_*	SS*_B_*	WT*_T_*	WT*_B_*	MWR*_T_*	MWR*_B_*	Drying Time
	(m^2^·°C/W)	(m^2^·Pa/W)	(0–1)	(0–1)	(mm/s)	(mm/s)	(s)	(s)	(mm)	(mm)	(min)
Mass (g/m^2^)	0.42	0.61 *	0.21	−0.40 *	0.44 *	−0.22 *	−0.3 *	0.36 *	0.36 *	−0.1	0.35 *
Thickness (mm)	0.63 *	0.68 *	0.54 *	−0.07	0.09	−0.21 *	−0.04	−0.06	−0.03	−0.24 *	0.41 *
Bulk density (g/cm^3^)	−0.56 *	−0.4	−0.69 *	−0.33 *	0.45 *	0.2 *	−0.38 *	0.17	0.42 *	0.29 *	−0.2
Air permeability (mm/s)	0.31	0.25	0.35	0.5 *	−0.32 *	0.04	0.25 *	−0.23 *	−0.25 *	0.03	0.03
Porosity (%)	0.23	0.17	0.34	0.42	−0.55 *	−0.22	0.47 *	−0.21	−0.55 *	−0.42	0.3

**Table 3:** Mean value (SD) of moisture management properties measured on the top (T) and bottom (B) of fabrics: wetting time (WT), absorption rate (AR), maximum wetted radius (MWR), spreading speed (SS), and calculated accumulative one-way transport capability (R).

Fabric ID	WT*_T_*	WT*_B_*	AR*_T_*	AR*_B_*	MWR*_T_*	MWR*_B_*	SS*_T_*	SS*_B_*	R
	(s)	(s)	(%/s)	(%/s)	(mm)	(mm)	(mm/s)	(mm/s)	(%)
1. 70% PES + 30% CO	14.3	3.8	5.9	33	0	15.0	0.0	2.8	406.7
	(3)	(0.6)	(1.2)	(2.8)	(0.0)	(0.0)	(0.0)	(0.2)	(24.6)
2. 60% MAC + 38% CO + 2% ANTS	13.8	9.1	48.6	101.1	15.7	15.7	1.2	1.5	269.8
	(6.5)	(3.9)	(9.1)	(26.3)	(3.5)	(1.9)	(0.6)	(0.5)	(104.2)
3. 45% CO + 55% PES	3.2	5.6	44.0	48.5	20.0	15.0	3.6	2.1	−156.6
	(0.2)	(0.3)	(1.4)	(2.3)	(0.0)	(0.0)	(0.1)	(0.1)	(6.8)
4. 50% AR + 50% CV FR	120.0	5.1	0.0	55.0	0.0	5.0	0.0	1.0	926.2
	(0.0)	(1.4)	(0.0)	(7.4)	(0.0)	(0.0)	(0.0)	(0.2)	(74.7)
5. 50% AR + 50% CV FR	5.2	5.5	26.3	45.1	17.0	18.0	2.5	2.4	172.5
	(0.7)	(0.8)	(5.2)	(10.1)	(2.7)	(2.7)	(0.4)	(0.4)	(62.5)
6. 50% PES + 50% CV	15.4	10.7	56.2	72.6	13.9	14.4	0.8	0.9	270.6
	(4.5)	(3.2)	(18.6)	(23.7)	(4.2)	(3.0)	(0.4)	(0.3)	(63.5)
7. 100% PES	13.3	6.4	12.2	45.2	2.9	18.6	0.4	2.5	291.4
	(7.9)	(2.4)	(9.4)	(21.8)	(7.6)	(6.3)	(1.1)	(1.1)	(146.2)
8. 86% PES + 14% EL	2.6	2.6	51.1	64.0	25.0	24.0	5.4	5.4	174.9
	(0.1)	(0.1)	(4.8)	(2.5)	(0.0)	(2.2)	(0.2)	(0.3)	(33.8)
9. 56% CMD + 40% PA + 2% CF + 2% EL	4.1	10.3	34.4	39.5	21.5	15.0	4.6	2.4	−75.8
	(1.3)	(3.1)	(11.1)	(13.5)	(2.4)	(3.3)	(0.6)	(1.2)	(27.1)
10. 68% CV FR + 29% AR + 2% EL + 1% ANST	5.2	5.1	35.5	59.5	20.0	19.3	2.8	2.6	253.1
	(1.1)	(0.6)	(12.3)	(4.8)	(0.0)	(1.9)	(0.2)	(0.3)	(48.4)
11. 100% CO	7.3	4.8	12.7	29.3	14.4	15.0	1.9	2.3	175.8
	(4.2)	(0.5)	(11.0)	(9.8)	(1.8)	(0.0)	(0.7)	(0.1)	(25.0)
12. 50% CO + 50% rPES	64	6.7	8.9	17.5	6.0	12.0	0.9	1.8	77.4
	(59.1)	(2.2)	(12.5)	(15.9)	(9.7)	(7.5)	(1.5)	(1.4)	(60.5)
13. 50% CO + 50% rPES	120	8.7	0.0	78.0	0.0	5.0	0.0	0.6	1010.0
	(0.0)	(1.4)	(0.0)	(9.2)	(0.0)	(0.0)	(0.0)	(0.1)	(20.9)
14. 100% PES + graphene print	11.0	4.9	7.0	31.1	5.6	20.6	0.6	3.8	300.5
	(4.1)	(2.0)	(2.9)	(3.1)	(10.5)	(1.8)	(1.4)	(0.5)	(41.8)
15. 80% WO + 20% PA	27.5	7.1	28.0	66.6	15.0	15.0	0.7	1.1	474.4
	(20.5)	(1.2)	(15.7)	(3.6)	(4.5)	(4.5)	(0.4)	(0.4)	(208.1)
16. 45% CO + 55% PES	85.9	1.9	7.9	61.9	3.0	6.0	0.1	2.5	1188.2
	(41.8)	(0.4)	(8.2)	(5.1)	(2.7)	(2.2)	(0.1)	(0.5)	(109)
17. 95% CO + 5% CF	120	3.2	0.0	40.3	0.0	5.0	0.0	2.1	1180.7
	(0.0)	(3.0)	(0.0)	(23.9)	(0.0)	(0.0)	(0.0)	(1.0)	(110.3)
18. 100% AR	3.6	4.0	39.8	63.3	21.0	21.0	3.8	3.6	230.6
	(0.9)	(1.1)	(5.0)	(7.1)	(2.2)	(2.2)	(0.7)	(0.6)	(24.9)

**Table 4:** Spearman’s correlation coefficients for drying time and thermal properties. Numbers in bold with * represent significant results (*p*-value < 0.05).

	Ret	Rct	SS*_T_*	SS*_B_*	WT*_T_*	WT*_B_*	MWR*_T_*	MWR*_B_*
	(m^2^·Pa/W)	(m^2^·°C/W)	(mm/s)	(mm/s)	(s)	(s)	(mm)	(mm)
Drying time (min)	0.63 *	0.58 *	−0.14	−0.59 *	0.47 *	0.17	−0.14	−0.65 *

## Data Availability

The original contributions presented in this study are included in the article. Further inquiries can be directed to the corresponding author.
